# The KCNE genes in hypertrophic cardiomyopathy: a candidate gene study

**DOI:** 10.1186/1477-5751-10-12

**Published:** 2011-10-03

**Authors:** Paula L Hedley, Ole Haundrup, Paal S Andersen, Frederik H Aidt, Morten Jensen, Johanna C Moolman-Smook, Henning Bundgaard, Michael Christiansen

**Affiliations:** 1 Department of Clinical Biochemistry and Immunology, Statens Serum Institut, Copenhagen, Denmark; 2 Department of Biomedical Sciences, University of Stellenbosch, Cape Town, South Africa; 3 Department of Internal Medicine, Roskilde Hospital, Roskilde, Denmark; 4 Department of Bacteriology, Statens Serum Institut, Copenhagen, Denmark; 5 Department of Medicine B, Rigshospitalet, Copenhagen, Denmark

## Abstract

**Background:**

The gene family *KCNE1-5*, which encode modulating β-subunits of several repolarising K^+^-ion channels, has been associated with genetic cardiac diseases such as long QT syndrome, atrial fibrillation and Brugada syndrome. The minK peptide, encoded by *KCNE1*, is attached to the Z-disc of the sarcomere as well as the T-tubules of the sarcolemma. It has been suggested that minK forms part of an "electro-mechanical feed-back" which links cardiomyocyte stretching to changes in ion channel function. We examined whether mutations in KCNE genes were associated with hypertrophic cardiomyopathy (HCM), a genetic disease associated with an improper hypertrophic response.

**Results:**

The coding regions of *KCNE1*, *KCNE2*, *KCNE3*, *KCNE4*, and *KCNE5 *were examined, by direct DNA sequencing, in a cohort of 93 unrelated HCM probands and 188 blood donor controls.

Fifteen genetic variants, four previously unknown, were identified in the HCM probands. Eight variants were non-synonymous and one was located in the 3'UTR-region of *KCNE4*. No disease-causing mutations were found and no significant difference in the frequency of genetic variants was found between HCM probands and controls. Two variants of likely functional significance were found in controls only.

**Conclusions:**

Mutations in KCNE genes are not a common cause of HCM and polymorphisms in these genes do not seem to be associated with a propensity to develop arrhythmia

## Background

Hypertrophic cardiomyopathy (HCM) is a condition characterised by increased wall (predominantly septal) thickness, diastolic dysfunction, and an increased risk of heart failure, stroke and cardiac arrhythmia [1]. The disease has a prevalence of 1:500 in young adults [2], and is considered a hereditary disease caused by mutations in more than 12 genes [3], most of which encode proteins of the sarcomere. The disease exhibits considerable intra-allelic as well as phenotypic heterogeneity. Presently, a genetic aetiology can be identified in 70% of familial cases and 30% of non-familial cases [3].

Recently, mutations in genes coding for ion channels have been shown to cause cardiomyopathy. Mutations in *SCN5A*, coding for the α-subunit of the ion channel conducting the depolarising *I*_Na _-current [4,5], and in *ABCC9 *[6], coding for the cardiac specific SUR2A subunit of the K_ATP _potassium channel, have been associated with dilated cardiomyopathy (DCM). The DCM caused by mutations in both *SCN5A *and *ABCC9 *is accompanied by cardiac arrhythmia.

The *KCNE*-gene family (*KCNE1*-*5*) encodes five small single transmembrane peptides (minK and MiRP1-4, respectively) that function as β-subunits to potassium and pacemaker ion channels [7,8]. The KCNE peptides confer distinctive characteristics to a variety of currents [9-11]. For example, the slow increase and high conductance characteristic of I_*Ks*_is conferred by minK (encoded by *KCNE1*) to the α-subunit (encoded by *KCNQ1*) [12]. The KCNE peptides are also involved in correct trafficking of α-subunits [13]. Mutations in *KCNE *genes have been associated with a number of diseases, i.e. cardiac arrhythmia by mutations in *KCNE1 *(long QT syndrome and Jervell Lange Nielsen Syndrome) [14-17], *KCNE2 *(long QT syndrome, atrial fibrillation, drug induced ventricular fibrillation) [18-20], *KCNE3 *(Brugada syndrome) [21] and *KCNE5 *(atrial fibrillation) [22]; mutations in *KCNE3 *have also been associated with periodic paralysis and hypo-and hyperkalemic disorders [23]. Furthermore, *kcne2 *null mice develop rhythm disturbances [24] and *kcne2 *null pups to *kcne2 *null dams develop hypertrophy among other abnormalities as a consequence of hypothyroidism [25]. This suggests that in addition to the development of arrhythmias, mutations in *KCNE2 *could give rise to cardiac hypertrophy through the dysregulation of thyroid hormones. Likewise, other investigations using *kcne2 *null mice have revealed an association with gastric pathology [26]. These finding suggest that the KCNE genes may influence phenotypic presentation of HCM in multiple ways.

All *KCNE *genes are expressed in the heart but to a varying extent [27]. The minK and MirP peptides exhibit considerable functional promiscuity, consequently, they may substitute for each other with different α-subunits [28] and the relative levels of peptides in different parts of the heart influence the regional variation of ion channel function [27].

Yeast-two-hybrid (Y2H) experiments have shown that minK is linked to the z-disc of the sarcomere via interaction with titin-cap (telethonin) [29]. The link between the T-tubule, where minK is attached and the Z-disc, has been suggested to constitute a "mechano-electrical feed-back system", linking the function of repolarising ion channels to stretch of the cardiomyocytes [29].

The Z-disc proteins are involved in the control of cardiac hypertrophy as mutations in the protein constituents of the Z-disc, T-cap, titin, muscle LIM protein, actinin and cypher/ZASP, have been shown to cause both HCM and DCM [30,31]. The electrical remodelling seen in heart failure is characterised by a marked increase in the expression of KCNE1 [32] in the heart.

We hypothesised that variants in *KCNE *genes, might result in changes in mechano-electrical feed-back, and could be responsible for a maladaptation of the stretch-response of the heart. This could explain an exaggerated hypertrophic response and thus HCM development in patients with mutations in Z-disc proteins. Alternatively, an increased occurrence of electrophysiologically significant *KCNE *variants might explain the increased propensity of arrhythmia in HCM.

We screened the genes *KCNE1, KCNE2, KCNE3, KCNE4*, and *KCNE5 *for genetic variants in 93 unrelated probands with HCM and related the findings to occurrence of disease or propensity to a particular phenotype.

## Results

No putative disease causing mutations were found in HCM index patients in any of the five *KCNE *genes. Fifteen genetic variants were identified; four of which were previously unknown. Fourteen of the genetic variants were located in the coding regions of the genes. The variants are detailed in Table 1. All variants were in Hardy-Weinberg equilibrium, when the variants were so frequent that this could be assessed.

**Table 1:** Genetic variants within the *KCNE *genes identified in a Danish HCM cohort

nucleotide	peptide	rs#	Pop rare allele frequency	HCM rare allele frequency	Disease association	Reference
KCNE1: [NM_000219.2/NP_000210.2]						

**c.24 G > A**	p.A8A		0.000	0.005		

**c.112G > A**	p.G38S	rs17846179	0.494	0.376	AF	[40]

**c.253G > A**	p.D85N	rs1805128	0.012	0.005	IVF, drug induced	[18,35,36]

KCNE2: [NM_172201.1/NP_751951.1]						

**c.22A > G**	p.T8A	rs2234916	0.043	0.005	IVF, drug-induced	

KCNE3: [NM_005472.4/NP_005463.1]						

**c.2T > C**	p.M1T		0.003	0.000		

**c.198T > C**	p.F66F	rs2270676	0.104	0.080		

**c.248G > A**	p.R83H	rs17215437	0.003	0.011	Hypokalemia	[7,41]

KCNE4: [NM_080671.2/NP_542402.2]						

**c.69C > T**	p.S23S	rs12720447	0.011	0.006		

**c.81C > T**	p.G27G	rs3795886	0.730	0.717		

**c.264T > C**	p.P88P	rs10201907	0.949	0.933		

**c.422A > C**	p.E141A		0.003	0.000		

**c.435T > G**	p.D145E	rs12621643	0.712	0.724		

**c.471G > A**	p.E157E		0.042	0.023		

c.*19G > C	3'UTR	rs10189762	0.059	0.046		

KCNE5: [NM_012282.2/NP_036414.1]						

**c.97C > T**	p.P33S	rs17003955	0.206*	0.150*	AF	[22,42]

* gender corrected value						

Two variants, p.M1T in *KCNE3 *and p.E141A in *KCNE4*, were found in single controls. The p.M1T variant abolishes the translation initiation codon and most likely results in haplo-insufficiency. The p.E141A variant, affects an amino acid which is conserved in seven species, and represents a charge change and may well modify the functional properties.

Some of the identified variants have previously been associated with arrhythmia, i.e. p.S38G in *KCNE1 *and p.P33S in *KCNE5*, that are known polymorphisms associated with increased risk of atrial fibrillation. There was no significant difference in the frequency of any of the polymorphisms between HCM and the normal population. Two variants, i.e. p.D85N in *KCNE1 *and p.T8A in *KCNE2 *have previously been associated with increased risk for drug-induced ventricular fibrillation. For both variants, the frequency was lower in HCM, for p.D85N rare allele frequency 0.5% vs 1.2% in controls, and for p.T8A a rare allele frequency of 0.5% vs. 4.3% in controls. For both variants the allele frequency was so low, however, that the difference is not significant when compensating for multiple comparisons.

The p.R83H variant in KCNE3 has previously been associated with hypo- and hyper-kalemia and paralysis [7], and here it was found in two cases. In one family the mutation was co-inherited with a mutation in troponin T and in another the comprehensive sarcomeric gene screening had not revealed other mutations. There were no special clinical characteristics of the carriers of the p.R83H variant. However, the p.R83H has, following the association with hypo- and hyper-kalemic paralysis, been described as a polymorphism in several populations [33].

None of the identified variants had any significant effect on splicing, i.e. did not interfere *in silico *with ESEs or SSEs.

## Discussion

The *KCNE *genes do not, despite the association with electromechanical feedback, seem to cause HCM, even though the number of probands examined does not preclude an involvement at the level of less than 1%. However, except in special cases, there does not seem to be any reason for including *KCNE *gene screening in the screening of genes in the genetic work-up of HCM.

The frequency of arrhythmia associated genetic variants was so low that it did not convincingly differ from that of controls and it cannot explain the increased occurrence of arrhythmia in HCM [34]. However, the previously arrhythmia-associated variants p.D85N [18,35,36] and p.T8A [7] both occurred more frequently in controls than in HCM patients. We cannot exclude, however, that a small minority of HCM patients with arrhythmia associated variants in *KCNE *genes have an increased propensity for arrhythmia.

The finding of very rare genetic variants with likely functional significance, i.e. p.M1T in *KCNE3 *and p.E141A in *KCNE4*, in controls is interesting and suggests that such variants may contribute to the arrhythmia risk in various conditions in the general population.

## Conclusions

Our findings suggest that neither *KCNE1*, despite its physical association with the Z-disc [29], nor the other *KCNE *genes are common causes of HCM.

## Methods

### Patients

Ninety-three unrelated consecutively diagnosed HCM patients identified at, or referred to, Copenhagen University Hospital, Rigshospitalet, Copenhagen, Denmark were included in the study. All patients were of Northern European descent. Patients were subjected to a full clinical evaluation including family history, physical examination, echocardiography and ECG. All fulfilled classical diagnostic criteria for HCM [37,38]. The mean age of index patients was 49 years, 62% were male, and 48% were familial. Ninety-two % had septal hypertrophy, 6% apical hypertrophy and 2% mid-ventricular hypertrophy. All patients had been screened for mutations in the coding regions of *MYH7, MYBPC3, TTNT2, TPM1, TNNI3, MYL3, MYL2, ACTC, TCAP, CSRP3*, and exons 3,7,14,18, and 49 of *TTN*, as detailed in a previous study [3]. All index patients were also screened for mutations in *GLA*. In 32 index patients this screening had identified presumably disease-causing mutations, i.e. 12 in *MYH7*, 8 in *MYBPC3*, 2 in each of *TNNT2*, *TNNI3 *and *GLA*, 1 in each of *ACTC*, *TPM1*, *MYL3 *and *MYL2*. Two patients were carriers of mutations in both *MYL2 *and *MYH7*. A control panel of 188 (50% men) anonymous blood donors obtained from Rigshospitalet, Copenhagen, were used.

### Molecular genetic studies

Genomic DNA was isolated from whole blood samples (Qiagen, Hilden, Germany). The genomic sequences of *KCNE1*, *KCNE2*, *KCNE3*, *KCNE4*, and *KCNE5 *were used for designing intronic primers covering the coding region of the genes. Primers and conditions are given in Table 2. DNA sequencing was performed using Big Dye technology. Variant numbering was verified using the Mutalyzer program [39].

**Table 2:** Primer sequences, amplicon size and melting temperatures for the *KCNE *gene mutation screening

Name	Sequence	amplicon size (bp)	Tm (°C)
KCNE1.1F	5' GCA GCA GTG GAA CCT TAA TG 3'	225	58
		
KCNE1.1R	5' CGG ATG TAG CTC AGC ATG AT 3'		

KCNE1.2F	5' CTT CGG CTT CTT CAC CCT G 3'	250	58
		
KCNE1.2R	5' TTA GCC AGT GGT GGG GTT C 3'		

KCNE2.1F	5' TCC GTT TTC CTA ACC TTG TTC 3'	250	58
		
KCNE2.1R	5' GCC ACG ATG ATG AAA GAG AAC 3'		

KCNE2.2F	5' GAT GCT GAG AAC TTC TAC TAT G 3'	300	58
		
KCNE2.2R	5' GTC TGG ACG TCA GAT GTT AG 3'		

KCNE3F	5' GCT AAG ATT TTA CCT GGG ATC TGA 3'	626	65
		
KCNE3R	5' TAT GCA CAA GGC TTC GGT CTA C 3'		

KCNE4F	5' CTC TTG TCA GCT GTT TGG CGA ACC 3'	886	65
		
KCNE4R	5' CAC AGG CAC CTC CCG GAC TC 3'		

KCNE5F	5' CCG CCG TGT CAC TCC CCG AAA 3'	493	62
		
KCNE5R	5' AGA TGA GGA GGG CGC GAA CCA 3'		

### Disease-causation and association

Genetic variants were considered disease-causing if 1) the nucleotide variation was deduced to result in a missense mutation, frameshift and/or abnormal splicing; 2) if relevant, the variation affected a conserved amino acid; 3) the variation co-segregated with the disease in affected family members and; 4) if the variation was not identified among 188 ethnically controlled samples. In the absence of available family members for co-segregation studies, disease association was presumed if criteria 1, 2 and 4 were fulfilled. If the mutation had previously, in accordance with the criteria mentioned here and/or relevant functional studies, been associated with disease, disease causation was presumed when just the criteria 1 and 4 were met. The association between gene variants and disease was assessed by comparing the distribution of variants in disease group and controls. χ^2^-testing was used to examine for significant association using a level of significance of 0.05, with correction for multiple comparisons, if such were made.

### Bioinformatics

ESE/SSE-*in silico *assessment was performed using the online web-servers: FAS-ESS [34], RESCUE-ESE [35], HMMgene [36], GENSCANW [37] and ESEFinder v.3.0 [38]. Multiple species alignments were performed using ClustalW2 [39].

### Ethics

Informed consent was obtained from study participants. The study was approved by the Local Science Ethics Committees, Copenhagen and Frederiksberg, protocol no. KF V92213.

## List of abbreviations

DCM: dilated cardiomyopathy; HCM: hypertrophic cardiomyopathy; Y2H: yeast-two-hybrid.

## Competing interests

The authors declare that they have no competing interests.

## Authors' contributions

PLH, PSA, JMS and MC Participated in the study design, PLH carried out the molecular genetic studies, PLH and FA participated in the sequence alignment and bioinformatics assessment of variants, PLH and MC drafted the manuscript, OH, MJ and HB Performed clinical characterisation of the patients, MC: Conceived the study.

All authors read and approved the final manuscript.
